# Are There Betel Quid Mixtures Less Harmful than Others? A Scoping Review of the Association between Different Betel Quid Ingredients and the Risk of Oral Submucous Fibrosis

**DOI:** 10.3390/biom12050664

**Published:** 2022-05-02

**Authors:** Nicola Cirillo, Peter Hung Duong, Wee Teng Er, Casey Thao Nhi Do, Manikkuwadura Eranda Harshan De Silva, Yining Dong, Sok Ching Cheong, Elizabeth Fitriana Sari, Michael J. McCullough, Pangzhen Zhang, Stephen S. Prime

**Affiliations:** 1 Melbourne Dental School, The University of Melbourne, Carlton, VIC 3053, Australia; pduong1@student.unimelb.edu.au (P.H.D.); w.er@student.unimelb.edu.au (W.T.E.); caseythaonhi.do@student.unimelb.edu.au (C.T.N.D.); m.desilva@student.unimelb.edu.au (M.E.H.D.S.); nancy.dong@student.unimelb.edu.au (Y.D.); m.mccullough@unimelb.edu.au (M.J.M.); 2 Translational Cancer Biology, Cancer Research Malaysia, 1 Jalan SS12/1A, Subang Jaya 47500, Selangor, Malaysia; sokching.cheong@cancerresearch.my; 3 Dentistry and Oral Health, La Trobe Rural Health School, La Trobe University, Bendigo, VIC 3550, Australia; e.sari@latrobe.edu.au; 4 School of Agriculture and Food, Faculty of Veterinary and Agricultural Sciences, The University of Melbourne, Parkville, VIC 3052, Australia; pangzhen.zhang@unimelb.edu.au; 5 Centre for Immunology and Regenerative Medicine, Institute of Dentistry, Barts and the London School of Medicine and Dentistry, Queen Mary University of London, London E1 4NS, UK; stephensprime@gmail.com

**Keywords:** betel quid, betel inflorescence, areca nut, stem quid, areca alkaloids, polyphenols, oral submucous fibrosis

## Abstract

Oral submucous fibrosis (OSF) is a potentially malignant condition of the oral cavity characterized by progressive fibrosis of the submucosal tissues. OSF is typically associated with the use of betel quid (BQ), a chewing package made of natural products (e.g., areca nut, betel leaves), with or without smokeless tobacco. BQ ingredients contain pro-carcinogenic bioactive compounds, but also potentially protective biomolecules, and we have shown recently that the chemical properties of different BQ recipes vary, which may explain the unequal prevalence of OSF and oral cancer in BQ users in different geographical regions. Hence, this scoping review was aimed at evaluating the existing literature regarding different BQ compounds and their association with OSF. The repository of the National Library of Medicine (MEDLINE/PubMed), medRxiv databases, Google scholar, Baidu scholar, CNKI, and EBSCO were used to search for publications that investigated the association between BQ chewing and OSF up to November 2021. The search terminology was constructed using the keywords “betel quid” and “oral submucous fibrosis”, and their associated terms, with the use of Boolean operators. The search was conducted under Preferred Reporting Items for Systematic Reviews and Meta-Analyses extension for Scoping Reviews (PRISMA-ScR) guidelines, together with clear inclusion and exclusion criteria. The review showed that the risk of developing OSF varied between different BQ recipes, and that chewing BQ mixtures containing betel inflorescence (BI) significantly increased the risk of OSF, as did the addition of tobacco. Conversely, the use of betel leaf in the mixture was likely to be protective, which may be due to the presence of polyphenols. Although further research is needed to determine the effect of individual BQ ingredients in the development of OSF, our pilot results provide the scope and rationale for informing future chemopreventive strategies for OSF and oral cancer in BQ chewers.

## 1. Introduction

Globally, approximately 600 million people chew betel quid (BQ). Three fourths of its consumption is concentrated in the Pacific Islands, South Asian, and Southeast Asian countries [1]. BQ chewing is typically associated with malignant and potentially malignant conditions of the oral cavity (OPMD), including oral submucous fibrosis (OSF), leukoplakia, erythroplakia, and lichen planus [2,3,4]. OSF is a chronic, oral potentially malignant condition characterized pathologically by juxta-epithelial inflammation, fibro-elastic change in the lamina propria, and epithelial atrophy, all of which led to progressive rigidity of the oral mucosa. The disorder commonly affects any part of the oral cavity and sometimes can include the pharynx [1]. OSF is a major global health issue because of its high malignant transformation rate, subsequent high mortality, and diminished quality of life [5]. In a recent meta-analysis, the pooled proportion of malignant transformation was 4.2% (95% CI: 2.7–5.6%) whilst the annual transformation rate was 0.73% [6]. Symptoms of OSF include xerostomia, recurrent ulceration, burning mouth sensation, and restricted mouth opening [7]. These factors influence a patient’s dietary intake, their ability to maintain oral health, and the capacity of clinicians to examine for oral cancer development.

The pathogenesis of OSF is complex and involves inflammation, hypoxia, and the generation of reactive oxygen species [reviewed in 2]. Although OSF is multifactorial in origin, there is strong evidence that areca nut (betel nut) chewing is the main etiological factor. Areca nut (AN) has been classified as an independent group 1 human carcinogen and is invariably chewed as a betel quid (BQ) in the Asia-Pacific region as well as in Asian migrant communities throughout the world [8,9]. Whilst not all BQs share the same constituents, the usual ingredients consist of AN, betel leaf (BL), catechu, and slaked lime, with or without tobacco. Variations are also known to exist including the addition of betel (*Piper betle*) stem and inflorescence (BS and BI), and various additives [10]. Traditionally, the four major areca alkaloids—arecoline, arecaidine, guvacoline, and guvacine, are regarded as potentially responsible for OSF and oral carcinogenesis. Mechanistically, biologically active AN alkaloids stimulate fibroblasts to increase collagen synthesis and due to decreased activity of collagenases, there is also reduced collagen degradation. These changes result in fibrosis of the underlying submucosa and are further associated with chronic inflammation and hypoxia [11]. Intriguingly, our preliminary observations suggest that chewing BQ from certain areas of Indonesia is associated with a reduced risk of OSF [12], thus prompting the need to further investigate the effect of different variations of BQ.

The aim of the present study is to systematically evaluate the existing literature regarding different BQ ingredients and their association with the onset of OSF. We believe that our findings, if developed further, will have important public health implications for the prevention of OSF and oral cancer.

## 2. Materials and Methods

### 2.1. Protocol and Search Strategy

The protocol for this structured scoping review was done in accordance with the Preferred Reporting Items for Systematic Reviews and Meta-Analyses extension for Scoping Reviews (PRISMA-ScR) guidelines [13]. This study was completed in order to identify the association between different BQ compositions and OSF as described in current literature. A search was conducted from all available literature up to the end of November 2021 using PubMed, medRxiv, Google Scholar, Baidu Scholar, CNKI, and EBSCO as the databases, and the keywords used were “Betel quid”, “Oral submucous fibrosis”, and their associated terms (Table 1). Appropriate sources of gray literature were also included in the search. The electronic searches were supplemented by a hand search of the bibliographies of all included articles.

### 2.2. Inclusion and Exclusion Criteria 

The articles that were included in this study had the following criteria: (1) they were based on humans; (2) they were written in the English language; (3) the BQ habit was practiced with or without tobacco; (4) the odds ratio (OR) or relative risk (RR), together with their corresponding 95% CI, were used as measures of an association between the BQ habit and OSF. Articles were excluded for the following reasons: (1) studies involved non-human samples; (2) studies were meta-analyses, systematic reviews; (3) studies in which an OR measured the association between BQ habit and oral potentially malignant diseases, and did not mention OSF exclusively. No time restriction was applied to the search. 

### 2.3. Study Selection and Data Extraction

The selection process was divided into three phases. In the first phase, the inclusion and exclusion criteria were used to select appropriate papers. In the second phase, papers were reviewed based only on their titles and abstracts under the guidance of the senior author (NC). Adhering to the Cochrane guidelines, a lenient policy was adopted during the selection process where there was any uncertainty. In the third phase, the full texts of publications meeting the inclusion criteria were analyzed. In cases of disagreement or ambiguity, a meeting was held to discuss the decision of inclusion, with the senior author (NC) to determine the final outcome due to seniority and expertise in the field. At the end of the three phases, the papers fulfilling all of the inclusion criteria were included in the qualitative analyses.

The following parameters were extracted from each study: name of the first author, year of publication, presence of tobacco, areca nut, betel leaf, slaked lime or other ingredients in the BQ mixture, type of study, the region where the study was conducted, sample size, and the OR/RR.

## 3. Results

### 3.1. Overview of the Search Process

**Figure 1:**
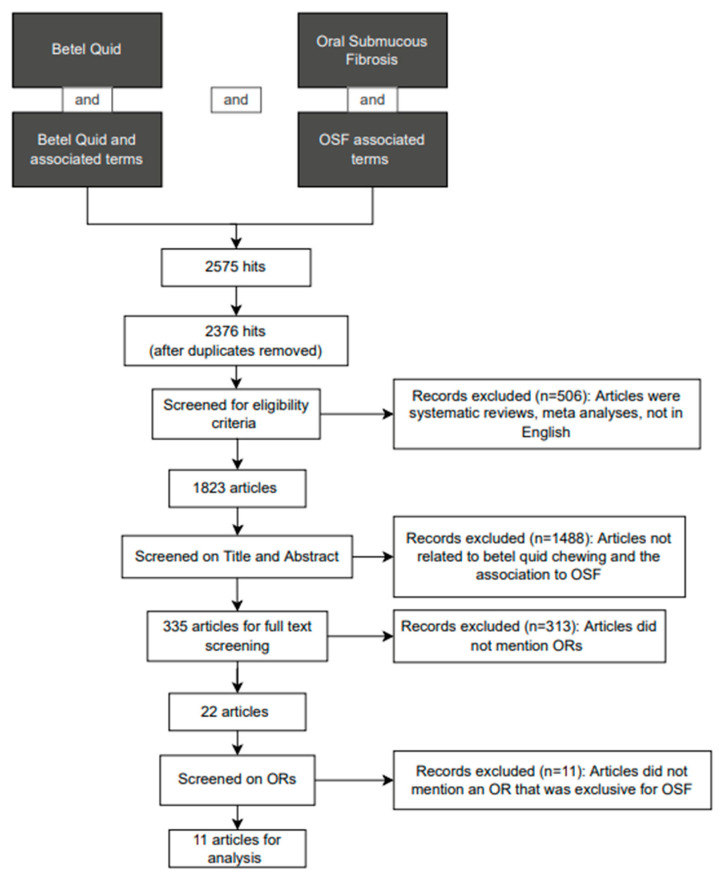
Flow chart of the selection process according to PRISMA guidelines. PRISMA, Preferred Reporting Items for Systematic Reviews and Meta-Analysis.

Details of the selection process are shown in Figure 1. A total of 2575 articles were retrieved with the search terms and using endnote and manual screening, 199 duplicates had to be removed. After applying the inclusion and exclusion criteria, there were 1823 articles, of which 335 remained after screening solely for the title and abstract. After reading the full text and appraising the articles, 313 articles were excluded as they did not meet all of the inclusion criteria, or they encompassed exclusion criteria.

**Figure 2:**
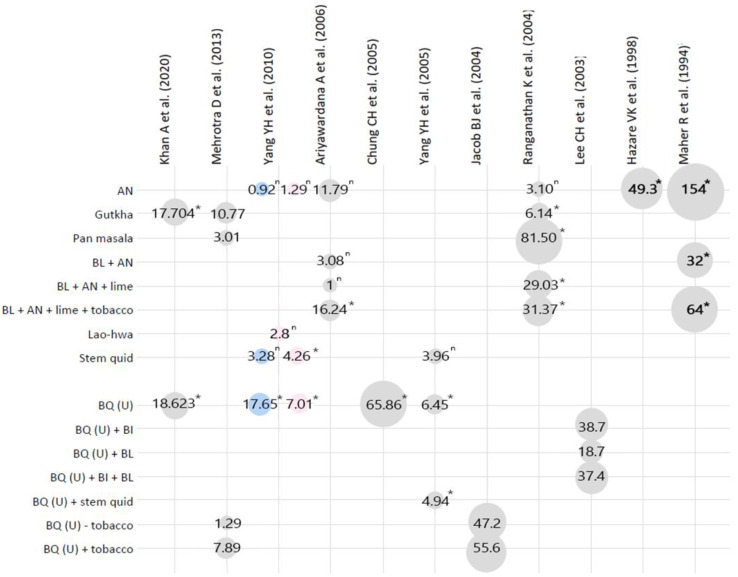
Overview of betel quid mixtures and their associated OR or RR values for OSF. ^n^, not significant to the 0.05 power. *, *p*-value < 0.05. Pink, females; Blue, males; grey, both genders. AN = areca nut; BL = betel leaf; BQ (U) = betel quid unspecified mixture; BI = betel inflorescence.

Of the remaining 22 articles, 11 papers were excluded because they reported ORs that were not exclusive to OSF; the remaining 11 papers were deemed to be eligible for detailed assessment (Figure 1). An overview of the BQ mixtures and their association with OSF is shown in Table 2. The number of participants in the included studies ranged from 102 to 49,085 with median 1007. ‘Unspecified’ is used to denote BQ mixtures where the exact components of the BQ were not specified in the article. The relationship between BQ and additives to OSF risk is shown in Figure 2.

### 3.2. Areca Nut 

Hazare et al. showed that the RR of OSF increased significantly with increased frequency of chewing AN; the consumption of AN at any frequency increased the risk of OSF development by approximately 50 times (RRany-frequency = 49.3; *p* < 0.01, RRno-AN-use = 1.0; *p* < 0.01) [23]. These results were further assessed by Maher et al. who showed that the RR of chewing AN was significantly higher than chewing pan masala and pan masala with tobacco (RR = 154, (95%CI: 34–693; *p* < 0.0001)); further, males had a higher risk than females (RRmales = 136 (95%CI: 7–2477; *p* < 0.001); RRfemales = 61 (95%CI: 14–262; *p* < 0.0001)) when comparing AN chewers with non-AN chewers [24].

Variations of these findings have also been reported. Ariyawardena et al. reported that chewing AN alone had a higher but not significant risk of developing OSF (OR = 11.79, (95% CI: 0.64–217.21; *p* = 0.058)) [17]. Yang et al. showed that OSF developed whilst chewing AN, but found that females had a higher risk than males in developing OSF (ORmales = 0.92 (95%CI: 0.10–8.12; *p* = 0.0114); ORfemales = 1.29 (95%CI: 0.29–5.80; *p* = 0.0143)) [16]. By contrast, Ranganathan et al. found that AN chewers had a lower risk of developing OSF compared to chewers who had AN with another additive such as pan masala, but this finding was not significant (OR = 3.10 (95%CI: 0.83–11.65; *p* = 0.078)) [21]. 

### 3.3. Pan Masala 

Pan masala consists of a combination of unripe AN, catechu, and slaked lime, with the potential for the addition of flavors and spices [8]. Notably, there is an absence of BL, BI, or betel stem in this formulation. In the papers analyzed in the present study, this variant of so-called ‘betel quid’ was present in Indian populations and only two articles described pan masala.

Mehrotra et al. reported that the OR of OSF with pan masala use was 3.01 (95%CI: 1.23–7.36) [15], whereas Ranganathan et al. recorded an OR value of 81.5 (95%CI: 4.95–1341.12) following the use of pan masala [21]. However, there was a significant difference between these two studies, which was the sample size, with Mehrotra et al.’s study having 3136 participants whereas Ranganathan et al.’s had 370 participants [15,21]. Overall, both studies demonstrated a significant positive association between pan masala consumption and increased risk of OSF.

### 3.4. Gutkha 

Gutkha is similar to pan masala; it has the same ingredients but with the addition of powdered tobacco and the use of ripe AN [8]. The three articles that described gutkha usage were from the Indian subcontinent. It appears that its practice is increasing and it is being used as a substitute for other BQ forms [14]. 

Khan et al. reported that OSF associated with smokeless tobacco was most prevalent and that gutkha was the most commonly used smokeless product (26.3%). Khan et al. reported an odds ratio of 17.704 (95% CI: 4.85–64.58) for developing OSF with a *p*-value < 0.001 [14]. These findings were confirmed by Mehrotra et al. who showed that gutkha usage was associated with an OR of 10.77 (95% CI: 8.18–14.18) [15]. In addition, Ranganathan et al. reported a statistically significant OR of 6.14 (95%CI: 1.77–21.32) in an Indian cohort of subjects [21]. 

Taken together, the data indicate that the use of a tobacco-containing smokeless product is associated with an increased likelihood of developing OSF. 

### 3.5. BQ and BQ (Unspecified) 

Ariyawardana et al. analyzed the BQ mixtures with BL and AN, and identified an OR of 3.08 (95% CI: 0.31–30.36) for developing OSF with a *p*-value of 0.62 [17]. Additionally, Maher et al. found an RR of 32 with a *p*-value < 0.05 [24].

For BQ mixtures without tobacco, Ariyawardana et al. reported an OR of 1 (95%CI: 0.24–4.16), indicating no statistically significant difference between the control group and BQ mixture (BL + AN + slaked lime) group [17]; by contrast, Ranganathan et al. reported a stronger, significant association of 29.03 (95%CI: 1.71–492.17) [21]. In the three studies which analyzed BQ mixtures with tobacco, they all showed a strongly positive and significant association with OSF [17,21,24]. For example, Ariyawardana et al. reported an OR of 16.24 (95%CI: 5.88–44.86) [17], Ranganathan et al. reported an OR of 31.37 (95%CI: 1.86–529.88) and Maher et al. reported the RR of 64 (95%CI: 15–274) [24].

For the unspecified BQ mixtures, 13 datasets of OR/RRs were available and the majority of studies were mainly undertaken in India and Taiwan. Although the mixtures were unspecified, it is important to note that most BQ mixtures from India contain tobacco whilst BQ mixtures from Taiwan mainly consist of AN, slaked lime, and piper BI with or without BL. Among the 13 datasets, 1 set reported a weak association between unspecified BQ and OSF [15], 4 sets reported a strong association [15,16,19], and 8 sets reported a very strong association [14,16,18,20,22]. 

### 3.6. Stem Quid 

Stem quid is a variant of quid in Taiwan and is a combination of unripe AN, BS, and slaked lime [8]. Yang et al. reported an OR of 3.96 (95% CI: 0.79–19.69) in 2005, whilst a study in 2010 reported an OR of 3.28 (95% CI: 0.55–19.55) in males and 4.26 (95% CI: 1.70–10.65) in females [16,19]. However, the 3.28 OR value was not significant (*p* > 0.05); this could be due to the smaller sample size in male stem quid chewers (*n* = 13). 

### 3.7. Lao-Hwa 

Lao-hwa is another variant of BQ that is used in Taiwan. It has a similar composition to BQ except that it uses BI instead of BS [8]. Yang et al. reported an OR of 2.8 (95% CI: 0.74–10.54) in females but this finding was not significant (*p* > 0.05); there was no OR value computed for males in this study, possibly due to the insufficient sample size [16].

## 4. Discussion

The aim of this scoping review was to examine whether there was an association between different BQ ingredients and the development of OSF. Tobacco has been confirmed to be associated with a significantly increased risk of OSF development. Pan masala, which does not contain BL, BI, BS, or lime had the second highest RR, and AN alone was reported to have the highest RR for development of OSF. The results also suggest that the use of BI is associated with higher risk of developing OSF, whereas BL might reduce the risk when added to AN. The addition of BL, BI, BS, and/or lime to AN would require more research to determine its mechanism and association with OSF development. 

The majority of studies in this report showed that BQ mixtures containing tobacco had increased risk for OSF compared to controls [15,17,20,21]. Maher et al. also reported a higher RR value with the addition of lime and tobacco (RR = 64) when compared to the mixture of BL and AN (RR = 32) [24]. It is important to note that smoked tobacco (e.g., cigarette) also increases the risk of OSF when associated with BQ chewing compared to BQ chewing alone [22]. The findings confirm previous observations that tobacco in smoked or chewed forms significantly increases the risk of OMPD, including OSF development [25,26]. Tobacco is a known carcinogen and it has been suggested that nitrosamines found in tobacco-products largely contribute to its genotoxic effects [25]. Tobacco undergoes metabolic activation by P450 enzymes to form N-Nitrosonornicotine (NNN), a known carcinogen that causes DNA damage leading to the development of potentially malignant diseases and, ultimately, oral cancer. In patients with OSF, progression to cancer may be related to smoking and alcohol, but also involves fibrosis-specific mechanisms. Interestingly, the primary genotoxic effect of smokeless tobacco appears to be due to the production of reactive oxygen species (ROS) and free radicals [25], and this same mechanism is also implicated in areca nut-induced oral cancer development [27]. Whether smokeless or chewed, tobacco could therefore exert similar pathogenetic mechanisms and increase the risk of OSF in BQ chewers regardless of the form in which it is consumed. Interestingly, there is a high incidence of OSCC in the New Guinea population despite the BQ mixture within this region not containing any tobacco at all. As such, these data imply that it is likely for AN to have carcinogenic effects in the development of OSF and OSCC without the influence of tobacco. This is in agreement with previous studies reporting that BQ and AN are carcinogenic without tobacco [28].

Consumption of AN is the primary risk factor for development of OSF as this condition is rarely observed in non-AN chewers [29]. AN, with or without tobacco, is carcinogenic in humans and causes increased collagen synthesis and ROS production, together with DNA and fibroblast damage [25]. The major constituents of AN are the areca alkaloids (arecoline) which contribute to its toxicity [29]. Arecoline contributes to the characteristic fibrosis in OSF by increasing expression of TIMP-1 which increases proliferation of fibroblasts and collagen production [30]. Additionally, arecoline suppresses endothelial cell proliferation which induces reduced vascularity and therefore promotes a hypoxic environment that pre-disposes tissues to carcinogenesis [31,32,33]. However, other AN constituents, when combined with lime, may also generate ROS and cause oxidative DNA damage [32]. AN is prepared in a variety of ways and is consumed in both its unripe or ripe form. The unripe form is a characteristic of Taiwan, whereas most other countries use it in its ripe form. AN chewers from Taiwan had lower OR values (OR = 0.92 and 1.29) compared to similar studies conducted in other countries (OR = 11.79 and OR = 3.10) [16,17,21]. This trend is perhaps not surprising because the alkaloid content of AN increases as it ripens and matures [34], thereby making it more carcinogenic and important in the pathogenesis of OSF [35]. Caution should be exercised in the interpretation of these findings, however, because other studies have found that unripe AN is more carcinogenic than ripe AN [12], possibly due to contrasting methods of preparation, different growing methods, prediction of ripeness by color, composition, and types of AN [36].

The addition of slaked lime to BQ mixtures or AN alone has been shown to cause microabrasions, and to facilitate the diffusion of potent chemicals and irritants into the oral mucosa [36]. Slaked lime leads to an increase in cellular turnover, ROS production in response to an alkaline environment, and DNA and chromosomal damage [30]. When combined with lime, the arecoline and guvacoline in AN are being hydrolyzed to arecaidine and guvacine. It is generally accepted that arecaidine is more potent, cytotoxic, and mutagenic compared to arecoline in increasing OSF risk [34]. It might be expected, therefore, that the risk of OSF would be increased in BQ containing slaked lime but Ariyawardana et al. compared the OR values between mixtures with and without slaked lime, and rather surprisingly, found OR values of 1 and 3.96, respectively [17]. It is possible that this reflects the small sample size of the study (*n* = 74 OSF patients); 4 cases and 4 controls for the BQ mixture with lime, and 3 cases and 1 control for the BQ mixture without lime. 

Yang et al. showed that BQ mixtures containing BS (stem quid OR = 3.28 and 4.26) had a higher risk than mixtures containing BI (Lao-Hwa OR = 2.8) [16]. Further, BQ mixtures containing BL (OR = 18.7) have a lower risk for OSF than mixtures containing BI (OR = 38.7) or mixtures containing both BL and BI (OR = 37.4) [22]. BI contains high concentrations of phenolic compounds which have a dual effect: whilst commonly regarded as anti-oxidants, in certain environmental conditions, they can act as human carcinogens in both oral keratinocytes and oral fibroblasts [2,37,38,39]. Polyphenols such as flavonoid, catechin, and tannins have been implicated in the pathogenesis of OSF as they promote collagen crosslinking which make these fibers more resistant to collagenase degradation [40]. Interestingly, it has been reported that BL can have potential chemoprotective effects in animal models [40]. Despite the antioxidant, anti-inflammatory, anti-apoptotic, anti-cancer, and anti-microbial properties of *Piper betle* leaf [41], there are currently no studies which explicitly investigate the effect of BL on OSF development in humans or establish the role of BL in preventing carcinogenesis, and early promising data have not been followed up yet [42]. We are currently attempting to fill this gap and, as a part of this aim, have characterized the chemical constituents of BQ ingredients, including the leaf, from different geographical regions [36]. The possible protective effect of betel leaves relies on the observation that the leaf contains abundant phytochemicals, i.e., hydroxychavicol, which contributes to the antiproliferative efficacy of betel leaf extract [41]. 

Whilst BQ increases the risk of developing OSF (OR = 17.65 and 7.01) more than stem quid (OR = 3.28 and 4.26) [16], little is known about the role of BS in OSF development. More research, therefore, is required to explain the difference in OSF risk when BS is compared to BL and BI. Furthermore, studies by Yang et al. and Ranganathan et al. have shown that the addition of BL, BI or BS and lime to AN results in higher OR values compared to chewing AN alone; these findings suggest an additive effect of these components in increasing OSF risk [16,21]. 

Genetic susceptibility is also an important factor to take into consideration whilst analyzing the effect of different BQ mixtures on the development of OSF. As only a relatively modest percentage of BQ chewers develop OSF, this suggests that there is a genetic component to disease pathogenesis that may predispose the individual to developing OSF [38]. Suggested genetic polymorphisms of collagen, MMPs, TIMPs, and TGF-B1 have been proposed, however, further clarifications on the exact polymorphisms and their direct effect on the relationship with development of OSF is needed [38]. For future studies, it would be important to note the effect genetic susceptibility has on disease and account for this when discussing the effects of different BQ mixtures in different regions. 

### Limitations of This Review

There are various limitations in the studies that have been included in this report. In terms of internal validity, the Khan et al. report is a retrospective cohort study and may have been subjected to a selection bias of controls, a recall bias, and a misclassification or information bias [14]. Maher et al. may also have had selection bias because the clinical criteria that were used would have excluded early forms or impending OSF cases and may have resulted in missed data [24]. There may also have been a risk of introducing recall bias due to the uncertainties associated with response reliability with questionnaire-based studies, and the fact that the accuracy of recalls was not verified either by repeating the interviews or by using objective criteria to confirm an individual’s habits or health status. 

Apart from the various combinations of ingredients that can be chewed in BQ, the preparation of AN itself can also contribute to the pathogenicity of the quid. For example, it has been shown that the polyphenols content in AN decreases as the nut matures [43], that roasted nuts contain the highest tannin content, and that sun-drying of the constituents reduces the arecoline content more than soaking and boiling in water [44]. Of the 11 articles analyzed in this review, none of them reported how the AN was prepared. In addition, there are regional differences in the preparation of AN. Products from Darwin in Australia, for example, appear to contain increased concentrations of alkaloids compared to India and Papua New Guinea [45]. Therefore, the variable of interest (exposure to AN or to specific BQ ingredients) might not share the same chemical composition across different studies. 

We acknowledge that there may also be several limitations to the external validity of the present report. For example, cigarette smoking and alcohol drinking are independent risk factors for OSF [46], and these may have not been taken into account in the regression models of the observational studies that investigated the association between BQ chewing and OSF. In one example, alcohol drinking elevated the overall risk of malignant transformation of OPMD by 23%, but also triggered a higher risk in OSF (aHR = 1.62 (1.06–2.47)) [46]. The exclusion of studies which were not published in English may also have limited our search results and introduced bias by discounting otherwise key data. The present report of the relationship between different BQ mixtures and the risk of OSF yielded only 11 articles, making it challenging to draw meaningful conclusions, especially for studies with minimal data and small sample sizes. Additionally, the articles in this review were limited to subjects from certain geographical regions/ethnic groups and hence, may not be applicable to other populations. Specifically, the results were confined to five articles from India, one from Pakistan, four from Taiwan, and one from Sri Lanka, and this may have biased our results to these regions. Importantly, the present study did not contain any BQ mixtures derived from Indonesia where it has been reported that chewing BQ mixtures from certain areas of Indonesia is associated with a reduced risk of OSF [12]. These findings, therefore, warrant further investigation, particularly with respect to the composition and preparation of the BQ mixture. 

## 5. Conclusions and Future Directions

Despite its known potential to cause OSF and other detrimental health effects, BQ and AN chewing is common in the Asia-Pacific region. AN, like tobacco, is addictive [47], and BQ plays an important role in religious practices, cultural rituals, and social customs in parts of the Asia-Pacific region [9]. The eradication of this habit, therefore, may not be realistically achievable in the short term. However, it might be possible to identify mixtures that have a greater or lesser risk of OSF, and by altering the incidence of OSF through BQ regulation, mortality can be reduced. With this in mind, we undertook this pilot scoping review. While our study confirms that tobacco is associated with an increased risk of OSF development, we found evidence that betel (*Piper betle*) leaf, when added to the chewing mixture, may be protective. Furthermore, pan masala and AN alone are associated with significant risk of OSF development. 

Unlike tobacco, for which the WHO Framework Convention on Tobacco Control provides evidence-based policies for reducing tobacco use, no global policy exists for the control of BQ and AN use [47]. Similarly, strategies to promote cessation in users of BQ products are scarce, and available data show that quitting rates among users are relatively low [48]. It would be desirable to implement measures to reduce the demand for BQ and AN, perhaps by adapting strategies that have been applied to tobacco, such as MPOWER [49]. Measures to limit BQ and AN use include monitoring their use, implementing prevention policies, offering help to quit tobacco use, warning about the dangers of BQ and AN consumption, enforcing bans on advertising, promotion, and sponsorship of AN-containing products, and raising taxes on these products. 

In summary, our scoping review shed light on the heterogeneity of BQ, and identified mixtures and ingredients that are associated with higher or lower risk of developing OSF. Although this was a pilot study, there is clear scope and indications for further research related to the constituents of BQ and OSF risk. To date, there is little, if any work on the possible protective effects of BQ ingredients, and this study’s results could provide an alternative route for reducing the incidence of OSF and OSCC in BQ users. 

## Figures and Tables

**Table 1:** Search terms used for this review.

Categories	Associated Terms
“BETEL QUID”	Betel OR masala OR lao-hwa OR gutkha OR tobacco OR paan masala OR betel mixture OR *Piper betle* stem OR Areca nut OR Betel quid OR Slaked lime OR Betel leaf OR Catechu OR Betel Inflorescence
“ORAL SUBMUCOUS FIBROSIS”	Oral submucous fibrosis OR OPMD OR OSF OR OSMF OR premalignant lesion* OR premalignant condition* OR oral potentially malignant OR oral precancer* OR (idiopathic scleroderma of the mouth) OR juxtaepithelial fibrosis OR idiopathic palatal fibrosis OR sclerosing stomatitis

**Table 2:** Overview of articles that were eligible for analysis. Typical betel quid ingredients, when present in the mixture, are labeled with “X”.

Study Author/s (Year)	Study Population	Tobacco	Areca Nut	Betel Leaf	Slaked Lime	Other	Study Type	Region
Khan A et al. (2020) [14]	1007 (*n* = 73 OSF)	X	X	X	X		Retrospective cohort	South India
Mehrotra D et al. (2013) [15]	3136 (*n* = 448 OSF)	X	X	X	X	Catechu	Population-based case control study	Lucknow, India
Yang YH et al. (2010) [16]	2020 (*n* = 89 OSF)	X	X	X	X	Stem	Cross-sectional study	Taiwan
Ariyawardana A et al. (2006) [17]	148 (*n* = 74 OSF)	X	X	X	X		Cross-sectional study	Sri Lanka
Chung CH et al. (2005) [18]	1075 (*n* = 17 OSF)	X	X	X	X		Cross-sectional study	Southern Taiwan
Yang YH et al. (2005) [19]	102 (*n* = 62 OSF)	X	X				Case control study	Taiwan
Jacob BJ et al. (2004) [20]	49,085 (*n* = 170 OSF)	X	X	X	X		Case control study	Kerala, India
Ranganathan K et al. (2004) [21]	370 (*n* = 185 OSF)	X	X	X	X		Case control study	Chennai, South India
Lee CH et al. (2003) [22]	1095 (*n* = 94 OSF)	X		X		Betel inflor-escence	Case control study	Kaohsiung, Taiwan
Hazare VK et al. (1998) [23]	200 (all OSF)		X				Case control study	Nagpur, Maharash-tra
Maher R et al. (1994) [24]	314 (*n* = 157 OSF)	X	X	X			Case control study	Karachi, Pakistan

## Data Availability

Supplementary results are available within the article. All data items not included in the manuscript are available upon reasonable request to the corresponding author.
